# The Impact of Curcumin on the Inflammatory Profile of SW982 Cells in a Rheumatoid Arthritis Model

**DOI:** 10.1155/2022/1208970

**Published:** 2022-04-12

**Authors:** Kamil Więcek, Piotr Kupczyk, Grzegorz Chodaczek, Marta Woźniak

**Affiliations:** ^1^Department of Clinical and Experimental Pathology, Wroclaw Medical University, Marcinkowskiego 1, 50-368 Wroclaw, Poland; ^2^Bioimaging Laboratory, Łukasiewicz Research Network-PORT Polish Center for Technology Development, Stablowicka 147, 54-066 Wroclaw, Poland

## Abstract

Rheumatoid arthritis (RA) is one of the most prevalent autoimmune diseases, affecting approximately 1% of the total global population. Curcumin, a natural polyphenol is a substance that could potentially mitigate the course of this disease. To evaluate curcumin's anti-inflammatory impact on synoviocytes in the RA model, a set of experiments was conducted on SW982 cells, stimulated by IL-1*β*, IL-6, or TNF-*α* to emulate inflammation. During the research, the curcumin effect was evaluated by measuring cell survivability, expression of *MMP1* gene, subcellular localization of P70S6K1 protein, and its phosphorylated form and amount of produced IL-6 and TNF-*α*. Results of conducted experiments presented a positive impact of curcumin on synoviocytes in the RA model, by reducing SW982 cells' survivability, decreasing levels of *MMP1* gene expression and TNF-*α* protein production, which altogether confirm beneficial effects of the curcumin therapy in a RA *in vitro* model.

## 1. Introduction

Rheumatoid arthritis (RA) is one of the most widespread diseases in the form of chronic inflammation, affecting about 1% of the total global population, with yet not fully understood pathogenesis [1–4]. This autoimmune condition is characterized by various burdensome symptoms including pain, swelling, and stiffness in joints of hands, wrists, knees and feet, greatly reducing the comfort of life. Through RA development, consistent and self-perpetuating inflammation is established inducing joint damage beyond possible regeneration. Untreated progression of RA leads to heavy degradation of the cartilage and local bone tissue, generating advancing stiffness and local muscle atrophy, vastly reducing mobility [1, 5, 6]. Beyond causing damage to joints in nearly 40% of patients, rheumatoid arthritis may lead to the extra-articular manifestation. The most noticeable symptoms of those manifestations are rheumatic nodules; however, many others can induce various disorders, including diseases of lungs and blood vessels, anemia, peripheral neuropathy, and dire conditions in other tissues and organs [5, 7]. Other symptoms of RA not directly related to joints consist of fatigue, fever, and loss of weight. Progression of the disease with mentioned manifestations may eventually lead to permanent disability or even premature death [1, 6, 8].

Direct mechanisms of RA pathogenesis are mostly unknown and unravelling all potential causes of this disease is a complex and demanding task to do, due to its putative heterogeneity. RA seems to be more of a set of different, case-dependent pathological conditions, leading to common symptoms varying in course and severity between patients [9], thus making the development of personalized therapy even more tedious. The development of this disorder would not be possible without communication of immune cells with those localized within the joint, of which synoviocytes seem to play a crucial role [4].

Fibroblast-like synoviocytes (FLS) or simply synoviocytes are cells of mesenchymal origin building and orchestrating function of the synovial membrane. These cells are the main cellular fraction within the intimal layer of the synovium, where they are responsible for overseeing local homeostasis, regulating the flow of substances, and are essential for proper joint functioning. In addition to the fulfilling function of a physical barrier, synoviocytes also synthesize joint lubricants, regulate immunological processes, maintain extracellular matrix, and clear intra-articular debris [10].

During RA development, inflammation-related cytokines such as IL-1*β*, IL-6, or TNF-*α* activate FLS, changing their phenotype into the one resembling cancer (RA FLS). Cells lose anchorage dependence and contact inhibition and gain great proliferating and migratory capabilities leading to the formation of a tumor-like structure called pannus. RA FLS not only rapidly multiplicate but also release a wide range of substances which worsens the course of the disease. By releasing massive amounts of cytokines, chemokines, and adhesion molecules, activated synoviocytes promote the local influx of immune cells exacerbating autoinflammation. Furthermore, RA FLS are responsible for the destruction of joint tissues both directly and indirectly. Due to the production of diverse metalloproteinases (MMPs) and cathepsin K, they straightforwardly lead to the digestion of the cartilage and bone tissues. Additionally, they are the cause of chondrocyte activation, by expressing RANKL (receptor activator of nuclear factor kappa-ligand) and producing M-CSF (macrophage colony-stimulating factor), which stimulates osteoclastogenesis and leads to further degradation of mentioned tissues [7, 10–14]. For the aforementioned reasons, synoviocytes stand out as the key players in the development and severity of RA, thus making them a universal target for potential therapies. One of the possible treatments could involve the usage of substances of natural origin.

Curcumin, a natural polyphenol extracted from Curcuma longa, possesses plenty of proven properties, beneficial in ameliorating autoimmunological diseases, such as RA. Due to its structure, curcumin is a free radical scavenger, reducing redox-related inflammatory signaling [15]. Moreover, various researches showcased its ability to diminish other signaling pathways, including those related to the activity of NF-*κ*B, JAK-STAT, MAPK, or mTOR complex proteins [16–18]. Profitable attributes of curcumin have been shown to lay a heavy impact on the functionality of RA FLS by reducing their survivability and hence the number of overproliferating cells [19], decreasing expression of IL-1*β*, TNF-*α*, and COX-2 proteins and diminishing production of tissue destroying metalloproteinases (MMP-1, MMP-3, and MMP-13) [16, 20, 21]. Presented anti-inflammatory properties, safety, and low cost of usage make curcumin a great candidate for potential therapies, which are already tested in trials conducted on patients with RA [17, 19, 22]. Nonetheless, more experiments are still required to completely establish all of the curcumin's benefits in RA treatments.

In this study, properties of curcumin were evaluated by measuring its effects on the inflammatory profile of SW982 cells in the RA model, induced by synoviocytes stimulation with IL-1*β*, IL-6, IL-17, IL-23, and TNF-*α*. To assess the effects of curcumin on the chosen cell line, a series of experiments was conducted including MTT survivability assays, analysis of apoptosis with flow cytometry, measurement of *MMP1* gene expression via qRT-PCR, and evaluation of the mTOR pathway activity via P70SK61 and p-P70SK1 protein immunostaining. Additionally, multiplex analysis was performed to test curcumin's efficiency in reducing the expression of IL-6 and TNF-*α* in IL-17- and IL-23-stimulated cells.

## 2. Materials and Methods

### 2.1. Cell Culture

Synovial sarcoma SW982 (CLS Cell Lines Service GmbH, Germany) cells were cultured in DMEM-F12 medium with 10% FBS and 1% antibiotics. Culture reagents were bought from Gibco (Thermo Fisher Scientific Inc., Waltham, MA, USA). Cells were maintained at 37°C and 5% CO_2_ in a humidified atmosphere. For experiments, cells from the 2nd to 9th passages were used.

### 2.2. Preparation of Curcumin

Curcumin was bought from LKT Laboratories Inc., (USA). The purity was ≥97% of curcuminoid content. The 25 mM stocks of tested substance were prepared, dissolving in 1141.3 *μ*l of DMSO and freezing at -20°C.

### 2.3. Cell Viability Assay

The MTT assay is a colorimetric test used to measure cellular metabolic activity to indicate cell viability, proliferation, and cytotoxicity. In the MTT assay, metabolic active cells transform yellow tetrazolium salt MTT into purple DMSO-soluble formazan crystals. This process is possible due to the activity of mitochondrial dehydrogenase enzyme in living cells, which causes salt transformation. Cells were seeded at 8 × 10^3^ in 96-well culture plates, cultured for a day, and then incubated with cytokines (Peprotech, Rocky Hill, NJ, USA) at 50 ng/ml concentration or cytokines with curcumin for the next 24 hours, followed by the MTT assay. The MTT solution was added to wells at a final concentration of 1 mg/ml for 3 hours in an incubator. Next, formazan dye was solubilized with 50 *μ*l DMSO for 30 min. Absorbance was measured at 490 nm in a BioTek Well-plate Reader (Winooski, VT, USA). The control group absorbance was 100%, whereas treated samples' cell viability was counted using the formula: % = (*A* of experimental wells/*A* of the control wells) × 100%. After preliminary studies with different concentrations of curcumin, 5 and 10 *μ*M concentrations were chosen for further experiments.

### 2.4. Flow Cytometry-Apoptosis Assay

Cells were seeded at 200 × 10^3^ in 12-well culture plates and treated analogously to cells in the MTT assay; however, incubation with tested substances has been shortened to 6 hours. After the incubation, cells were collected from all wells and transferred to Eppendorf tubes. Next, cells were washed with PBS and centrifuged (6 min, 4°C, and 400 RCF) and suspended in 1 ml of binding buffer. Afterwards, 4 *μ*l Annexin V conjugated with FITC-A and 2 *μ*l 7-AAD were added to each sample, according to the manufacturer's instruction. Eppendorf tubes were vortexed and incubated without light for 30 min on ice. After the incubation, samples were analyzed with flow cytometry using the FITC channel for Annexin V and PC5.5 channel for 7-AAD (Cytoflex, Beckman Coulter Life Sciences, IN, USA). Negative samples were prepared without the staining and samples stained with one fluorochrome were used for compensation.

### 2.5. Cell Morphology Comparison

Cells were seeded and treated similarly to cells in the flow cytometry apoptosis assay. Photos were taken using the Olympus IX73 microscope with CellSens Programme (Olympus, Japan).

### 2.6. RNA Isolation, Reverse Transcription, and Real-Time PCR Gene Expression Analysis

Cells were seeded at 1 × 10^6^ in 6-well culture plates and treated analogously to cells in MTT assay. After incubation, total RNA was extracted using GeneMatrix Universal RNA Purification Kit (EURx, Poland). The quality and concentration of obtained RNA were evaluated using a NanoPhotometer® spectrophotometer (Implen GmbH, Germany). Next, 1000 ng of total RNA was reverse-transcribed on cDNA using smART First-Strand cDNA Synthesis Kit (EURx, Poland) according to the manufacturer's instructions using MJ Research PTC-100 thermocycler (Marshall Scientific, USA). Prepared cDNA was frozen at -20°C until next steps of gene expression analysis. Forward and reverse primer sequences for target and housekeeping gene were synthesized (Genomed, Poland) and used at 0.5 *μ*M concentration per reaction. The sequences are listed as follows: GAPDH forward 5′-AGCCACATCGCTCAGACAC-3′, GAPDH reverse 5′-GCCCAATACGACCAAATC-C-3′, MMP1 forward 5′-GCTAACCTTTGATGCT-ATAACTACGA-3′, and MMP1 reverse 5′-TTTGTGCGCATGTAGAATCTG-3′.

The real-time PCR reaction was performed using SG on Taq qPCR Master Mix (EURx, Poland) with Light Cycler 480 instrument and LightCycler®480 SW 1.5.1 software (Roche, Switzerland) in 50 cycles. Parameters of reaction are as follows: initial denaturation for 10 min at 95°C, denaturation for 10 sec at 95°C, annealing for 10 sec at 60°C, and extension for 10 sec at 72°C. Relative gene expression was calculated using the *ΔΔ*CT method. The experiments were conducted twice with minimum two samples.

### 2.7. Confocal Microscopy

Cells were seeded at 6 × 10^3^ in 96-well culture plates and treated analogously to cells in the MTT assay. After the incubation, cells were rinsed twice with warm PBS, fixed with 4% paraformaldehyde in PBS for 10 min at room temperature, and then rinsed again two times with PBS for 5 min. Next, cells were blocked with blocking serum (5% donkey serum, 3% BSA, 01% Triton X-100, 0.01% Tween 20, 0.01% DMSO, and 0.5 M glycine in PBS) for an hour at room temperature. Following serum removal, mouse anti-*α* S6 p70 (H-9) and anti-*α* S6 p-p70 (A-6) (Santa Cruz Biotechnology, Inc., USA) primary monoclonal antibodies in dilution 1 : 2500 (in blocking serum) were added to wells for overnight incubation at 4°C. Afterwards, primary antibodies were rinsed with PBS for 4 × 5 min and donkey anti-mouse secondary antibodies conjugated with DyLight-488 (Novus Biologicals, USA) in 1 : 500 dilution (in blocking serum) were added for 2-hour incubation at room temperature and no light. After the incubation, cells were rinsed with PBS for 4 × 5 min and DAPI (dilution 1 : 1000, Merck KGaA, Germany) counterstaining was performed for 10 min at room temperature. Finally, cells were rinsed with PBS for 3 × 5 min and the culture plate was imaged on a Zeiss Cell Observer SD confocal microscope (Carl Zeiss, Germany) using a 10x air objective (NA 0.30) and QImaging Rolera EM-C2 EMCCD camera. The laser lines used for excitation were 405 nm for DAPI (BP 450/50 emission filter) and 488 nm for target proteins (FE01-520/35 emission filter) with constant exposure time and other camera settings for all tested conditions. Further steps of image processing were performed using Fiji-ImageJ software (National Institutes of Health, Bethesda, MD, USA).

### 2.8. Multiplex Cytokine Assay

Cells were prepared according to the previously described protocol. After treatment, cell culture medium was collected, centrifuged, and kept frozen before the Multiplex analysis. For the 96-well plate, cytokine levels were detected using Magnetic Bead MILLIPLEX assay kit (Millipore, Merck, Darmstadt, Germany) and MAGPIX Multiplexing System (Millipore) following the manufacturer's protocol. Standard curves were prepared by making a serial dilution according to the instructions. Median fluorescence intensity (MFI) values for the analyte were converted into absolute concentration using a five-parameter logistic (5-PL) curve-fit generated by the MILLIPLEX® Analyst 5.1 software (Millipore), cytokine concentration results were expressed in pg/ml and normalized to control, presenting ratio of cytokines production. Results were analyzed with xPONENT4.2 and Milliplex Analyst 5.1 data analysis software (Millipore).

### 2.9. Statistical Analysis

Analysis between the groups was conducted using the nonparametric Mann–Whitney test for abnormally distributed data, a *p* value below 0.05 was considered significant. The PAST programme, version 4.05 was used for calculations.

## 3. Results

### 3.1. The Effects of Curcumin, IL-1*β*, IL-6, and TNF-*α* on SW982 Cell Viability Measured by an MTT Assay and Apoptosis Analysis

To evaluate the proper dose of cytokines and curcumin for further analysis, MTT assay and flow cytometry experiments were performed. First, curcumin, its DMSO vehicle, and cytokines alone were examined. Then, SW982 cells were incubated together with chosen dose of cytokines and curcumin.

**Figure 1:**
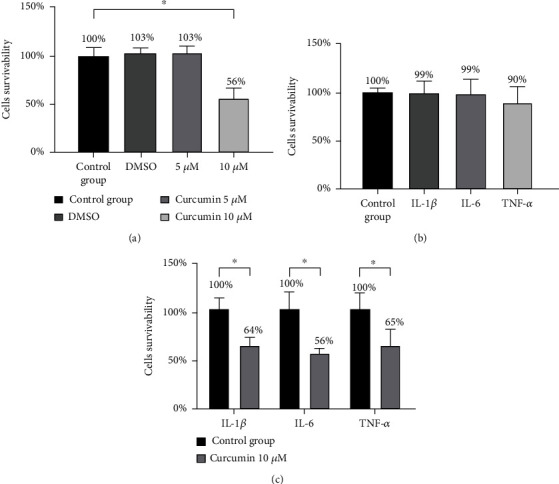
Comparison SW982 cell survivability after 24 h incubation with (a) curcumin (5 *μ*M, 10 *μ*M) and DMSO; (b) IL-1*β*, IL-6, and TNF-*α* at concentrations of 50 ng/ml; and (c) curcumin (10 *μ*M) and IL-1*β*, IL-6, and TNF-*α* (50 ng/ml). Results represent the mean from two different experiments from 5 wells, ^∗^*p* < 0.05.

The results indicated that neither incubation with cytokines at 50 ng/ml concentration, DMSO nor with curcumin at 5 *μ*M concentration caused any significant changes in cell survivability (Figures 1(a) and 1(b)). On the contrary, incubation with curcumin at the concentration of 10 *μ*M in both cytokine-stimulated and untreated groups killed cells, revealing cytotoxicity of 44%, close to the value of IC_50_ (Figures 1(a) and 1(c)).

**Figure 2:**
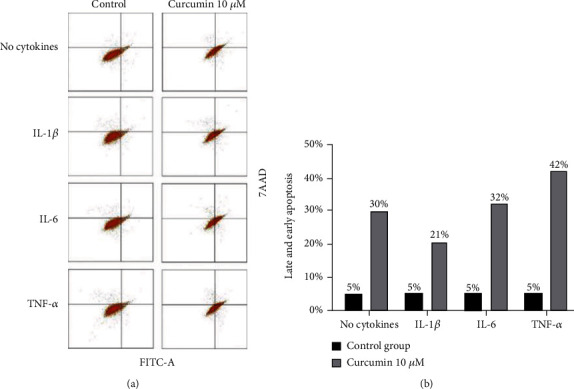
(a) Graphical presentation of representative data from flow cytometric analysis of apoptosis after 24 h cell incubation with curcumin (10 *μ*M) and cytokines (50 ng/ml). *x*-axis: intensity of FITC-A fluorescence. *y*-axis: intensity of 7-AAD fluorescence. Dot plots present alive cells: lower left; early apoptotic cells: lower right; late apoptotic cells: upper right; and dead cells: upper left. After treatment, the cells were stained using Annexin-FITC/7-AAD Kit and were measured by flow cytometry. (b) Bars represent the percentage of total apoptotic cells (early+late apoptosis) in cytokine-stimulated cells and control cells.

To confirm the results obtained in the MTT assay, analysis of apoptosis with flow cytometry was conducted. As shown in Figure 2, in all tested samples, the addition of curcumin caused serious differences in the sum of early and late cell apoptosis. This assay has proven that curcumin causes SW982 cell death mainly via the apoptotic pathway and that none of the used cytokines reduced cell survivability.

### 3.2. The Effects of Curcumin, IL-1*β*, IL-6, and TNF-*α* Coincubation on SW982 Cell Morphology

**Figure 3:**
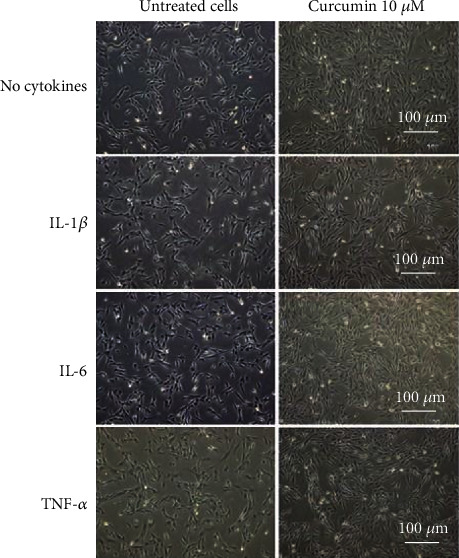
Representative images of SW982 cell morphology detected by phase-contrast microscopy after 24 h incubation with curcumin (10 *μ*M) and cytokines (50 ng/ml).

Incubation of synoviocytes with high concentrations of curcumin can change not only their functioning but also their morphology [16]. The effect of used treatment on SW982 cell morphology was evaluated by a series of photos captured with the usage of light microscopy. Obtained results did not present any noticeable differences between cells before and after incubation apart from an increase in number of round, bright dead cells (Figure 3). Similar observations were made through the course of all experiments.

### 3.3. The Impact of Curcumin on the Expression of MMP1 Gene in Inflamed SW982 Cells

**Figure 4:**
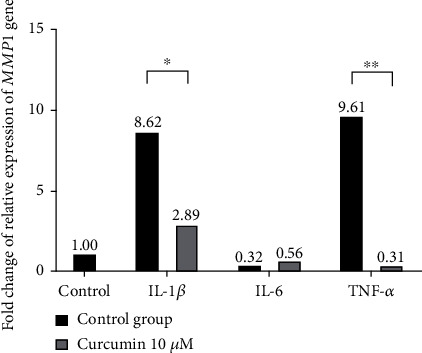
Representative image of gene expression analysis of MMP1 gene coding tissue destroying metalloproteinase performed by quantitative RT-PCR. Comparison of MMP1 gene expression in SW982 cells after 24 h incubation with curcumin (10 *μ*M) and different cytokines (50 ng/ml). Data are presented as representative calculation from two independent experiments, ^∗^*p* ≤ 0.05, ^∗∗^*p* ≤ 0.01.

To measure whether curcumin decreases the expression of the *MMP1* gene in SW982 cells in the RA model, qRT-PCR was performed. The results show that incubation with IL-1*β* and TNF-*α* significantly increased the relative expression of *MMP1* in SW982 cells. The addition of curcumin substantially decreased *MMP1* expression in IL-1*β* and TNF-*α* to a level below the control group. Relative gene expression of the tested gene in samples incubated with IL-6 was lower than in the control group, independently of curcumin addition (Figure 4).

### 3.4. The Impact of Curcumin of Subcellular Localization of P70S6K1 and p-P70S6K1 in Inflamed SW982 Cells

**Figure 5:**
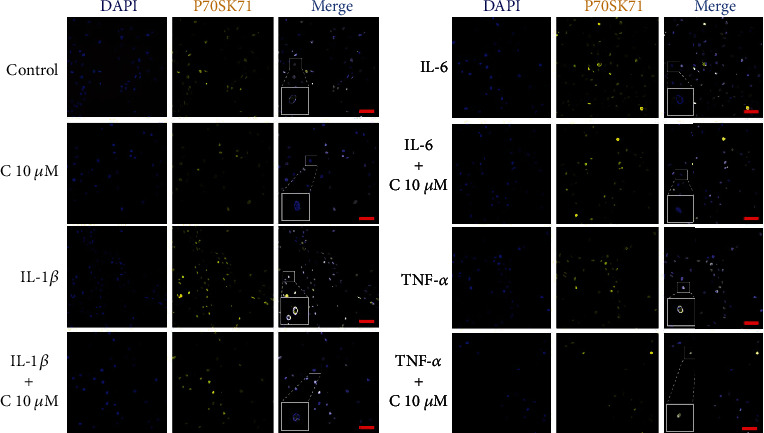
Representative images of subcellular localization of the P70SK61 protein in SW982 cells after 24 h incubation with curcumin (10 *μ*M) and cytokines (50 ng/ml). The photographs were taken on a Zeiss Cell Observer SD confocal microscope. Scale bar = 100 *μ*m.

**Figure 6:**
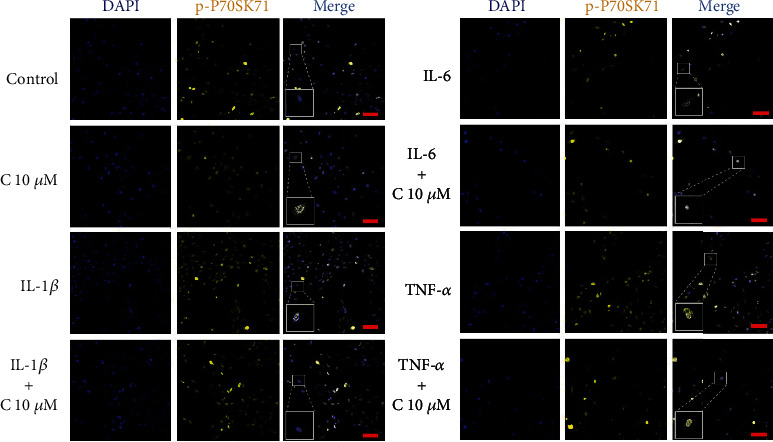
Representative images of subcellular localization of the p-P70SK61 protein in SW982 cells after 24 h incubation with curcumin (10 *μ*M) and cytokines (50 ng/ml). The photographs were taken on a Zeiss Cell Observer SD confocal microscope. Scale bar = 100 *μ*m.

**Figure 7:**
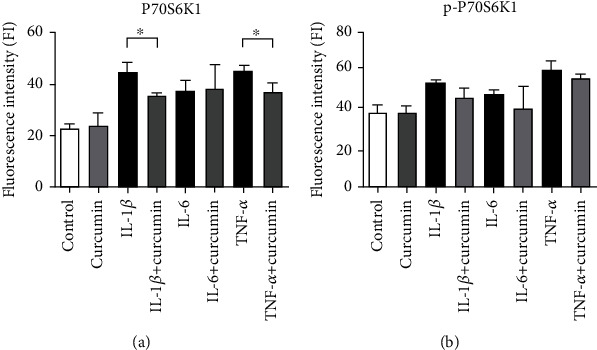
Bars represent densitometry analysis from confocal microscopy evaluation of P70S6K1 and its phosphorylated form. Statistical significance was measured by Dunn's test, ^∗^*p* ≤ 0.05, ^∗∗^*p* ≤ 0.01. Results indicated statistical significance between curcumin and IL-1 *β* and TNF- *α* SW982-treated cells in the levels of unphosphorylated protein.

To assess whether the addition of curcumin may lay an impact on the expression and subcellular localization of dephosphorylated and phosphorylated form of P70S6K1, the immunofluorescence staining procedure was conducted. P70S6K1 is kinase involved in ribosome activation and is a direct substrate of mTOR complex; thus, its concentration, level of phosphorylation, and subcellular distribution may indicate changes in activity of PI3K-Akt-mTOR pathway. Obtained results indicate no difference in the subcellular distribution of P70S6K1 and p-P70S6K1 in tested groups comparing to control (Figures 5 and 6). Further densitometric analysis shows that incubation of cells with cytokines increased the amount of analyzed proteins. Addition of curcumin led to slight, but statistically significant decrease of P70S6K1 levels in SW982 cells stimulated with IL-1*β* and TNF-*α*, but not of its phosphorylated counterpart (Figure 7).

### 3.5. Impact of Curcumin on the Expression of IL-6 and TNF-*α* in Inflamed SW982 Cells

**Figure 8:**
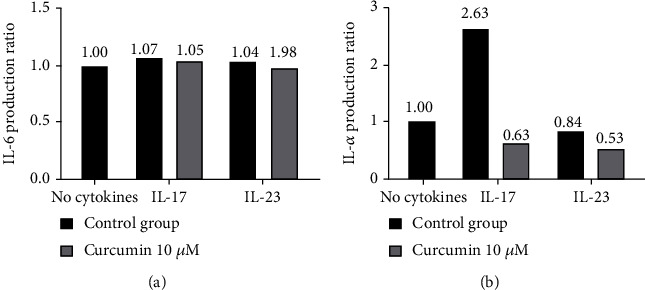
(a) Comparison of IL-6 production in SW982 cells after 24 h incubation with IL-17 or IL-23 with or without addition of 10 *μ*M curcumin measured by multiplex analysis. (b) Comparison of TNF-*α* production in SW982 cells after 24 h incubation with IL-17 or IL-23 (at 50 ng/ml) with or without addition of 10 *μ*M curcumin measured by multiplex analysis. Data are presented from one experiment conducted from two wells.

The effect of curcumin on the expression of chosen proinflammatory cytokines was measured by multiplex analysis of IL-6 and TNF-*α* production by SW982 cells. The results show that curcumin decreases significantly the production of TNF-*α* in IL-17- and IL-23-stimulated cells but did not change the expression of IL-6 (Figure 8).

## 4. Discussion

Rheumatoid arthritis is one of the most prevalent autoimmune diseases affecting millions of people all around the world. During the course of this illness, chronic inflammation induces numerous symptoms, which may in result greatly reduce the quality of life, causing permanent disability, or leading even to premature death. The pathophysiology of this disease presents extremely complicated network of different cells and mediators, making the development of new therapies a very demanding task. Nowadays, many researchers focus on finding new potential therapies for RA that will reduce the proinflammatory response of affected joints. Moreover, different *in vitro* and *in vivo* models of this disease allow scientists to follow detailed pathways and focus on the utilization of natural substances such as curcumin. The SW982 is a synovial sarcoma cell line widely used and accepted in experiments regarding rheumatoid arthritis. Due to its sarcoma characteristics, this cell line exhibits similar properties as synoviocytes in the state of developed inflammation. Although model made with the usage of primary cell cultures would be closer to the real-life situation, the SW982 cell line was chosen to avoid variation between samples and experiments.

In the present study, the effectiveness of curcumin in decreasing the inflammatory profile of activated synoviocytes has been investigated in the SW982 cell model of RA. The inflammation was induced by incubating cells with various proinflammatory cytokines including IL-1*β*, IL-6, IL-17, IL-23, and TNF-*α*. This in vitro model enabled the evaluation of curcumin effect on cytokine-stimulated cells.

The results of MTT assays and analysis of apoptosis indicated that 24-hour long incubation of synoviocytes with curcumin significantly reduced cell survivability, inducing apoptosis to a similar degree independently of used cytokines. Obtained data were similar to results obtained by Kloesh et al. [23] and Harati et al. [24] and suggested the potent activity of this natural polyphenol to reduce proliferation and induce cytotoxicity of activated by inflammation synoviocytes. This observation is also confirmed by Cai et al. [25] who demonstrated that the joint destruction by synoviocytes' massive proliferation can be inhibited by curcumin.

Incubation of cells with high concentrations of curcumin may change not only their functioning but also their morphology [16]. However, our experimental observations ruled out the impact of the tested polyphenol at the concentration of 10 *μ*M on changes in the SW982 cell appearance. This effect may be explained by the fact that the researchers used more than twice higher dose of curcumin in their studies. Moreover, they performed the experiments in a different cellular model based on human fibroblast-like synoviocytes (FLS) isolated from RA synovial tissues obtained at joint replacement surgery.

In the experiment conducted by Moon et al. [20], curcumin decreased the production of MMP-1 protein in FLS. This finding was later confirmed in the publication released by Dai et al. [26], who measured the amount of MMP-1 in the synovium of rats in the collagen-induced arthritis (CIA) model of RA. The qRT-PCR data obtained in this study indicated curcumin decreased the *MMP1* gene expression in SW982 cells stimulated with IL-1*β* and TNF-*α*, supporting the positive impact of the tested polyphenol on the inflammatory profile of activated synoviocytes.

In our work the levels of *MMP1* mRNA in groups incubated with IL-6 and with IL-6 in presence curcumin were substantially lower than in the control. These outcomes seem to corroborate the results obtained by Choi et al. [27], proving that IL-6 does not increase MMP-1 production in synoviocytes.

The mTOR or rather PI3K-Akt-mTOR is one of the major signaling pathway connecting cellular anabolisms with the cell's external surrounding, including the inflammatory milieu. Through the cascade started by the activation of PI3K by cytokines or PAMPs (pathogen-associated molecular patterns), mTOR complex leads to the activation of various molecular responses such as phosphorylation of P70S6K1 protein. The P70S6K1 is a kinase that upon activation (transition into p-P70S6K1 phosphorylated form) increases protein synthesis [28]. Due to its main function of ribosomal kinase, this protein is mostly localized within the nucleus compartment. Our results show that curcumin did not change the subcellular distribution of studied proteins *in vitro*. Additionally, the densitometry analysis showed that levels of P70SK61 in curcumin-treated cells decreased in comparison to SW982 stimulated by IL-1 *β* and TNF- *α*, but not by IL-6. At the same time p-P70S6K1 protein level in curcumin-treated cells remained similar to control cells treated with cytokines. Obtained results confirmed curcumin activity leading to decreased levels of P70SK61 in synovial cells. This was also demonstrated by Dai et al. [26], who evaluated the native kinase using CIA rat model with curcumin at a dose of 200 mg/kg. The lack of statistically significant differences in the p-P70S6K1 fluorescence intensity may indicate that curcumin did not inhibit phosphorylation of P70S6K1, but rather decreased its overall quantity, attenuating the mTOR pathway activity.

The last experiment was conducted to evaluate the impact of curcumin on the expression of IL-6 and TNF-*α* in IL-17- and IL-23-stimulated SW982 cells. The addition of curcumin decreased significantly the production of TNF-*α* but not of IL-6. Various publications confirmed the positive effect of curcumin in the reduction of IL-6 and TNF-*α* production in autoimmunological disorders including RA models [16, 26, 29, 30]. Our data based on Multiplex analysis results supported the curcumin's properties in reducing inflammation-related responses in activated synoviocytes by TNF-*α*. On the other hand, the effectiveness of curcumin to decrease IL-6 production was not statistically significant, which contradicts other published data possibly due to differences in used models and methods.

Despite promising properties in decreasing the inflammation in the cellular model of RA, curcumin possesses some qualities greatly reducing its potential application in therapies. Free curcumin is characterized by low water solubility and poor bioavailability and is rapidly metabolized and degraded after administration [31]. Those drawbacks could be potentially mitigated by using various carriers, for example in form of liposomes [23, 32] or solid lipid nanoparticles [33], which are still being under extensive research. The study bears several additional limitations, related to the low number of tested cytokines and the limited concentration range of used stimulants. The decrease of P70S6K1 level by curcumin is encouraging; however, more research is needed to fully evaluate the effect of this polyphenol on mTOR and other proinflammatory pathways at the molecular level. Further animal and clinical studies should include evaluation of curcumin's absorption, biodistribution, and tissue concentration in relation to the microenvironmental conditions (e.g., pH, inactivation by proteases in the synovial environment, and limited access to inflammatory cells within the articular tissue) providing information about its actual mode of action in vivo [34]. Such studies may not only expand our knowledge on curcumin activity but also, most importantly, give us better insight into mechanisms that exacerbate the inflammation and lead to the development of diseases like RA, increasing chances for the development of new therapies.

## 5. Conclusions

**Figure 9:**
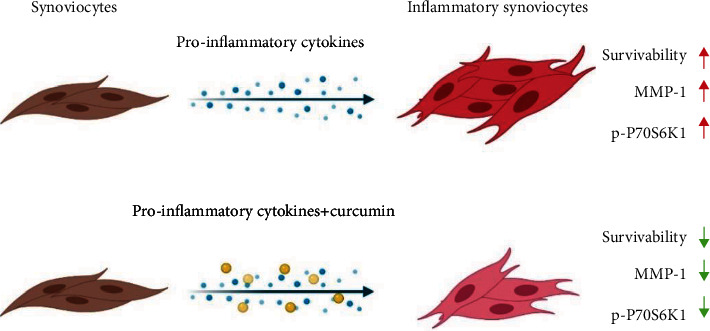
Graphical abstract presenting the curcumin effect of the inflammatory marker reduction in the proposed rheumatoid arthritis *in vitro* model.

The study evaluated the impact of curcumin on the inflammatory profile of stable SW982 cells in the in vitro RA models obtained with different proinflammatory cytokines. Aside from proving the overall anti-inflammatory properties of curcumin, results of conducted experiments showed some of the molecular basics of its action with emphasis on the mTOR signaling pathway, including visualization of P70S6K1 cellular distribution. Additionally, acquired data confirmed that IL-6 did not increase MMP-1 expression in fibroblast-like synoviocytes (Figure 9).

Conducted investigations indicate the promising role of curcumin in decreasing the inflammation, enhancing its position as the potential compound in potential RA therapies.

Nonetheless, it is crucial to continue investigations on the curcumin's impact on chosen detailed pathways involved in the RA pathogenesis in different models of RA. Hence, further research involving different doses of curcumin and *in vitro/in vivo* models would be required to fully evaluate the potential of this natural polyphenol in the RA treatment and confirm its efficacy as a natural carrier for potent therapy.

## Data Availability

Data are contained within the article.

## Additional Points

*Sample Availability*. Samples of the curcumin are available from the authors.
